# The Potential of DHA as Cancer Therapy Strategies: A Narrative Review of In Vitro Cytotoxicity Trials

**DOI:** 10.3390/nu15082006

**Published:** 2023-04-21

**Authors:** Jaqueline de Freitas Rodrigues, Hellen Kempfer Philippsen, Maria Fani Dolabela, Cleusa Yoshiko Nagamachi, Julio Cesar Pieczarka

**Affiliations:** 1 Institute of Biological Sciences, Federal University of Pará, Belém 66075-110, Pará, Brazil; 2 Socioenvironmental and Water Resources Institute, Federal Rural University of the Amazon, Belém 66077-830, Pará, Brazil

**Keywords:** fish oil, anticancer molecules, in vitro experiments, highest incidence cancer, pharmacotherapy

## Abstract

Docosahexaenoic acid (DHA), also known as omega-3 (*n*-3) polyunsaturated fatty acid (PUFA), is a natural compound that has demonstrated pharmacological activity against several malignant neoplasms. Available cancer treatments cause side effects, affect healthy cells, reduce the quality of life of patients and may cause resistance to antineoplastics. For these reasons, the search for new therapies is continuous. This narrative review aimed to compile information on in vitro experiments that study the cytotoxic effect of DHA or molecules derived from DHA in tumor and nontumor cells. This was performed to highlight the potential of DHA as a strategy for cancer therapy and to gather information, which will help researchers plan experimental designs and develop research to discover effective therapies against cancer. In addition, studies were presented that demonstrate the dose of DHA that can treat patients with cancer. Thus, a search was conducted for articles on the SCOPUS and Web of Science platforms, published until 2022, that analyzed the action of DHA against breast, lung, colorectal, prostate, stomach and liver cancers. Cytotoxic effects were observed in tumor and nontumor cell lines, and these results varied with the type of cell line studied, drug concentration, incubation time and treatment combination, i.e., with DHA alone, combined with other drugs and with molecules derived from DHA. In patients with cancer, in all analyzed studies, DHA intake was associated with eicosapentaenoic acid (EPA) and/or proteins to aid chemotherapy, and with this procedure, tumor reduction, chemotherapy tolerance and muscle mass gain were obtained. This work contributes to the community by demonstrating the possible applicability of DHA in the pharmaceutical area of oncological therapies.

## 1. Introduction

Cancer is a pathology with a silent onset and is characterized by the uncontrolled anticipation of malignant cells [1]. This disease affects thousands of people worldwide, and the number of new cases and deaths is growing every year [2,3]. In 2018, there were 18.1 million new cases of cancer and 9.6 million deaths [4]. In 2020, the estimated number of new cases was 19.3 million, and there were 10 million deaths [5].

In 2020, the types of cancer with the highest global incidence, considering both sexes, were breast (11.7%), lung (11.4%), colorectal (10.0%), prostate (7.3%) and stomach (5.6%), and those with the highest mortality were lung (18%), colorectal (9.4%), liver (8.3%), stomach (7.7%) and breast (6.9%) [5].

Currently, the drugs epirubicin, oxaliplatin, fluorouracil, cisplatin and capecitabine are used in cancer therapy, but they have not been internationally standardized for this treatment [6,7]. One of the principles of chemotherapy is cytotoxicity, which is the ability to kill cancer cells; however, cytotoxicity often affects healthy cells and causes side effects, which reduces the quality of life of patients. In addition, cancer is susceptible to becoming resistant to drugs [8,9,10]. Thus, finding efficient therapies to combat cancer is of great interest [11].

Docosahexaenoic acid (DHA) is an omega-3 PUFA with lipophilic characteristics [12,13]. The disposition and amount of unsaturation in DHA favor more potent biological activity and less unsaturation compared to that of other fatty acids; thus, DHA is susceptible to oxidative stress processes [14,15]. DHA helps in the prevention of cardiovascular diseases [16] and premature retinopathy [17] and promotes anti-inflammatory action [18] and anticancer activity [10].

A few years ago, some fatty acids were evaluated in the treatment of cancer, with emphasis on DHA treatments that show the potential to inhibit uncontrolled cell proliferation [18], increase the cytotoxic capacity of antineoplastic agents and which do not interfere significantly in the quality of life of people [19]. In cells, the entry of fatty acids occurs by rapid diffusion and through the support of membrane proteins, such as fatty acid transport protein (FATP), fatty acid binding proteins (FABP) and fatty acid translocase (FAT), which are also responsible for PUFA metabolism and activity [20,21,22]. In cancer cells, the modulation of these membrane receptors is high, and with the acidic tumor microenvironment, the excessive internalization of lipids in the cell and consequent development of lipid droplets inside may occur [23,24]. The accumulation of DHA inside cells can cause irreversible oxidative stress, generating ferroptosis, which consists of a type of nonapoptotic cell death that causes tissue destruction due to the biological dysfunction of proteins and cell membranes [25,26].

This narrative review aimed to compile information on in vitro experiments that study the cytotoxic effect of DHA or molecules derived from DHA in tumor and nontumor cells. This was performed to highlight the potential of DHA as a strategy for cancer therapy and to gather information to help researchers plan experimental designs and develop research to discover effective therapies against cancer. In addition, studies were presented that demonstrate the dose of DHA capable of treating patients with cancer.

For this, a search was carried out for articles available on the SCOPUS, Web of Science and PUBMED platforms, published until 2022. A total of 2422 articles were found available on the SCOPUS platform, 1709 on the Web of Science and 263 on PUBMED that related the keywords docosahexaenoic acid and cancer of the breast, lung, colorectal, prostate, stomach and liver, listed among the types of cancer with the highest incidence/mortality. From the articles found, this review included studies that performed in vitro cell viability assays of DHA or molecules derived from DHA in breast, lung, colorectal, prostate, stomach and liver malignant neoplasms, those that performed comparisons of these types of cancer with nontumor cells and those that analyzed the influence of DHA intake on the therapy of patients with these types of malignant neoplasms.

Based on this review, it was possible to relate the concentrations used to treat these types of cancer in in vitro cell viability assays, describe DHA activity in tumor and nontumor cell lines and present different DHA therapeutic strategies against this pathology.

## 2. Materials and Methods

This narrative review included experimental articles that described in vitro cytotoxicity assays in breast, lung, colorectal, prostate, stomach and liver cancer cell lines treated with DHA alone or combined with other drugs or encapsulated in nanocarriers or molecules derived from DHA. In addition, studies that used these treatments in comparative analyses of these types of cancer with nontumor cell lines were included. For this study, the SCOPUS, Web of Science and PUBMED platforms were searched without excluding the language, country or year of the article. The keywords and Boolean operators used were (1) “DOCOSAHEXAENOIC ACID” AND (“breast cancer”); (2) “DOCOSAHEXAENOIC ACID” AND (“lung cancer”); (3) “DOCOSAHEXAENOIC ACID” AND (“colorectal cancer” OR “colorectal cancer”); (4) “DOCOSAHEXAENOIC ACID” AND (“prostate cancer”); (5) “DOCOSAHEXAENOIC ACID” AND (“stomach cancer” OR “gastric cancer”); and (6) “DOCOSAHEXAENOIC ACID” AND (“liver cancer”). The reference list of the collected articles was also considered to find relevant articles. All studies identified through this search strategy were initially evaluated by reading the title, abstract, materials, methods and results described and presented in graphs. Articles were independently reviewed by two authors and discussed until a consensus was reached. In vitro studies were included if they met the following criteria: type of cytotoxicity assay, treatment incubation time, treated cell line and concentration of test substance. The criteria for studies that included in vivo doses of DHA were to be conducted in humans with the types of cancer that were part of this review. In tables, only the data of the articles that clearly and logically presented the identification of the *p* value in the graphs were included. When there was a statistically significant difference, the results in the tables are presented as follows: * *p* value < 0.05; ** *p* value < 0.01; *** *p* value < 0.001. The results with *p* values ≥ 0.05 were considered nonsignificant (ns: not significant). The other studies described in the results were included considering the percentage of cytotoxicity described by López-García et al., 2014, p. 3. Cellular inhibition below 20% is considered noncytotoxic; within 20–40% is weak; 40–60% is moderate cytotoxicity and over 60% is strong. All articles included in this review are shown in Table 1, with their respective year of publication, journal and impact factor.

## 3. Results

### 3.1. Treatment of Tumor and Nontumor Cell Lines with DHA Alone or Combined with Other Drugs or Encapsulated in Nanocarriers or with Molecules Derived from DHA

In vitro cytotoxicity studies on breast cancer cell lines are presented below; lung; colorectal; prostate; gastric; liver treated with DHA alone or combined with other drugs or encapsulated in nanocarriers or with molecules derived from DHA. These cancers are among the types with the highest incidence and overall mortality, available in the GLOBOCAN database [5]. Among the studies compiled in this review, there are also comparative analyses between tumor and nontumor cell lines. The results of this review described the types of in vitro assays performed, treatment time, treated cell lineage, the concentrations of DHA and other drugs tested and the results of the analyses demonstrated in the experimental articles.

#### 3.1.1. In Vitro Cytotoxicity Studies in Breast Cell Lines

Table 2 summarizes the data on the in vitro cell viability analysis in tumor and nontumor breast cancer cell lines treated with DHA alone, in association with other substances and with different molecules derived from DHA. The treatment with DHA was compared to apatinib in MDA-MB-231 cells using the Cell Counting Kit-8 (CCK-8) (Table 2) [38].

The cell viability of MDA-MB-231 cells was statistically decreased when treated with DHA in a concentration range from 25 µm to 800 µm and with apatinib in a concentration range from 12.5 µm to 200 µm, with inhibition in a dose-dependent manner, within 48 h. When MDA-MB-231 cells were treated with the combination of DHA and apatinib, there was also a significant decrease in cell viability (Table 2); however, it is possible to observe that the activity of the substances is lower when used alone than when combined. A study was carried out with several breast cell lines [42]. The lineages tested included normal breast basal epithelial cells (MCF-10F), normal transformed breast cells (trMCF) and breast cancer cells (bsMCF, MDA-MB-231, T-47D and SK-BR-3). These cell lines were treated with DHA and with the molecules derived from DHA, 4-hydroxyl-docosahexaenoic (4-OH-DHA) and 4-oxo-docosahexaenoic (4-OXO-DHA) for a period of 96 h at a concentration of 100 µM, and for analysis, the colorimetric assay 3-(4,5-dimethylthiazolyl-2)-2,5-diphenyltetrazolium bromide (MTT) was used. These authors also submitted MDA-MB-231 cells to treatments with these substances at concentrations of 10, 25, 50 and 100 µM within 96 h.

The nontumor lineage MCF-10F and trMCF showed statistically significant inhibition for the three substances tested at a concentration of 100 µM. Among the tumor cell lines tested at a concentration of 100 µM, the only cell line that did not show cytotoxicity was T-47D. In the test with different concentrations of DHA, 4-OH-DHA and 4-OXO-DHA, different results were obtained according to the substances tested. With DHA, cytotoxicity was significant at concentrations of 25, 50 and 100 µM; with 4-OH-DHA, at concentrations of 10, 25, 50 and 100 µM; and with 4-OXO-DHA, only at concentrations of 50 and 100 µM.

Assessments using the CellTiter 96^®^ AQueous One Solution kit assay were performed with tumor MDA-MB-231 and nontumor MCF-10F cell lines, using the substance derived from DHA, known as 13R,20-dihydroxydocosahexaenoic acid (13R,20-diHDHA), at concentrations of 1, 10, 20, 30 and 40 µM, for a period of 24 h [45] (Table 2). An in vitro analysis using the MTT assay was conducted comparing the cytotoxicity obtained with 4-OXO-DHA in the treatment periods of 24 h, 48 h, 72 h and 96 h in MDA-MB-231 cells [42] (Table 2).

In another study no statistically significant cell inhibition was observed at any concentration and in any cell line tested with 13R,20-diHDHA (Table 2) [45]. In an in vitro analysis, the following results were obtained: in the 24 h period, 4-OXO-DHA did not cause statistically significant cellular inhibition; within 48 h, inhibition occurred only at a concentration of 100 µM; in the period of 72 h and 96 h, cell inhibition occurred at concentrations of 50 and 100 µM (Table 2) [42].

In another recent study, which is not shown in the table, MTT assays were performed for the comparative analysis of the action of DHA and the drugs chloroquine and lovastatin in MDA-MB-231 cells after a 48-h incubation [29]. The tested concentrations were 25, 50, 75, 100, 200, 400 and 800 µM DHA; 3.75, 7.5, 15, 30 and 60 µM lovastatin; and 5, 10, 15, 20, 25 and 30 µM chloroquine (CQ). The results obtained revealed that DHA showed activity against breast cancer in a dose-dependent manner, with a percentage of cell inhibition that reached 56.96% at a dose of 800 µM. At a concentration of 25 µM, DHA showed no cytotoxic effect. In this study, the 50% inhibitory concentration of DHA obtained was 680 µM. Lovastatin showed inhibition of 29.11% at the lowest concentration and 49.13% at the highest. Chloroquine did not demonstrate cytotoxic activity against these cells [29].

After observing these results, DHA + LOVA, DHA + CQ, DHA + LOVA + CQ and LOVA + CQ were compared at concentrations of 100 µM (DHA), 30 µM (LOVA) and 20 µM (CQ). In this new analysis, the inhibition obtained with DHA associated with LOVA or CQ was greater than when each of these substances was used alone. DHA alone (100 µM) inhibited 24.32%, LOVA (30 µM) inhibited 40.37% and CQ (20 µM) showed no cytotoxic activity. However, when DHA was associated with LOVA, it inhibited 44.12%, and when associated with CQ, it inhibited 47.06%. DHA associated with CQ demonstrated a considerable change in the percentage of cytotoxicity. It was also observed that the combination of DHA + LOVA + CQ showed a 51.96% reduction in cell viability [29].

In another study, which evaluated the cytotoxic activity of DHA alone or associated with sorafenib, an antineoplastic agent, through a 3-(4,5-dimethylthiazol-2-yl)-5-(3-carboxymethoxyphenyl)-2-(4-sulfophenyl)-2H-tetrazolium (MTS) assay. Human breast cancer cell lines (MDA-MB-231 and MCF-7) were subjected to concentrations of 50 µM DHA, 0.1 µM sorafenib, 1 µM and 3 µM sorafenib and the same doses of these substances used in combination in the treatment of MDA-MB-231 and MCF-7 for a period of 48 h [34]. It was observed, in both lineages, that there was a synergistic action in the association of DHA with sorafenib, with cytotoxic activity in a dose-dependent manner. The MCF-7 line showed an inhibition percentage above 75%, which is a strong cytotoxic effect. In the MCF-7 lineage, the percentage of inhibition was higher than in MDA-MB-231. In another cytotoxicity analysis, Jiao et al., 2018 evaluated the concentrations of 25, 50 and 100 µM DHA in the presence of 0.5 µM sorafenib in MDA-MB-231 cells treated for a period of 24 h, 48 h and 72 h. In this analysis, DHA at concentrations of 50 and 100 µM, within 48 h of incubation, showed a moderate cytotoxic effect, with greater inhibition than at 24 h. At 72 h, the percentage of inhibition was even greater, and therefore, it is possible to consider an effect in a time–dose-dependent manner [34].

MTT assay was also used to evaluate DHA activity in MDA-MB-231 and BT-20 tumor cell lines [48]. For this analysis, the concentrations used were 10, 25, 50, 100 and 150 µM at 24 h and 48 h. BT-20 cells were more sensitive to the action of DHA than MDA-MB-231 cells. The cytotoxicity results found at 24 h were as follows: concentration of 10 µM, considered noncytotoxic; 25 µM, 23.13% reduction; 50 µM, 35.95%; 100 µM, 41.97%; 150 µM, 58.8% for BT-20 cells; concentrations of 10 and 25 µM, considered noncytotoxic; 50 µM, 24.43%; 100 µM, 35.78%; 150 µM, 52.12% for MDA-MB-231 cells. In the 48-h incubation period, the cytotoxicity results were as follows: 10 µM, 23.31%; 25 µM, 32.72%; 50 µM, 45.67%; 100 µM, 52.52%; 150 µM, 62.11% for BT-20 cells; concentrations of 10 and 25 µM, considered noncytotoxic; 50 µM, 36.4%; 100 µM, 57.68%; 150 µM, 67.28% for MDA-MB-231. These results demonstrate cytotoxicity in a dose- and time-dependent manner [48].

Nanotechnology was also used to analyze the in vitro cytotoxic effect of DHA against breast cancer. Nanotechnology is among the most modern drug approaches in the field of oncology [37]. The substances DHA, α-tocopherol succinate (TS) and doxorubicin (DOX) were carried in a nanostructured lipid carrier (NLC) to evaluate the cytotoxic activity in 4T1 cells. The assay used for this analysis was the sulforhodamine B-cell viability assay (SRB). Cells were exposed to the following concentrations and forms of treatment: NLC-DHA (0.29 µM, 1.16 µM, 4.5 µM, 9.07 µM), NLC-TS (0.16 µM, 0.63 µM, 2.44 µM, 4.88 µM) and NLC-DOX (0.04 µM, 0.16 µM, 0.62 µM, 1.25 µM) alone and the association of the three pharmacological substances in a 1:1 ratio for a period of 48 h [37]. NLC-DHA did not demonstrate cytotoxicity against breast cancer at the concentrations used. This same result was observed in relation to the NLC-TS. However, the treatment of 4T1 cells with NLC-DOX caused strong cytotoxicity starting at a concentration of 0.62 µM. A strong inhibitory effect was also observed in the association of NLC-DHA-TS-DOX at the two highest concentrations. With the results obtained, it was possible to observe that the percentage of cell inhibition was higher in therapy with NLC-DHA-TS-DOX [37].

#### 3.1.2. In Vitro Cytotoxicity Studies in Lung Cancer Cell Lines

Three studies that related the anticancer activity of DHA in lung cancer cell lines were selected and are shown in Table 3. In the study, through the MTT assay [36], human lung cancer cell lines A549 and H1299 were treated for 24 h with DHA concentrations of 10, 30 and 60 µM [36] (Table 3). In another study, human (A549 and H1299) and mouse (LLC) lung cancer cell lines were analyzed [17]. These cells were treated for a period of 24 h with DHA at concentrations of 25, 50, 75 and 100 µM (Table 3).

Treatment with DHA caused statistically significant cell inhibition at concentrations of 30 and 60 µM when applied to the A549 lineage. When applied to the H1299 cell line, there was only a reduction in viability at a concentration of 60 µM (Table 3) [36]. In another study, it was demonstrated, through the water-soluble tetrazolium (WST) assay, that DHA differentially reduced the viability of the three cell lines tested (A549, H1299 and LLC) (Table 3). In the A549 cells, the reduction in cell viability occurred at concentrations of 75 and 100 µM. In the H1299 and CLL cells, statistically significant inhibition occurred at doses of 50, 75 and 100 µM (Table 3) [17].

In another study, in vitro cell viability analysis was performed with different molecular species of DHA, DHA-enriched phosphatidylcholine (DHA-PC), DHA-triglyceride (DHA-TG) and DHA-ethyl esters (DHA-EE), which were applied to the human lung cancer cell line (95D). Yue et al. observed that DHA-PC significantly reduced cell viability at all concentrations tested (50, 100, 200 and 400 µg/mL) in all treatment periods (24 h, 48 h and 72 h) in a time–dose-dependent manner [47]. With regard to DHA-TG, at 24 h and 48 h, cell inhibition was considered statistically significant at concentrations of 100, 200 and 400 µg/mL, and at the 72-h treatment incubation time, there was a statistically significant reduction in all tested concentrations. In the treatment with DHA-EE, the authors observed that in the 24-h incubation period, only the highest concentration (400 µg/mL) caused a statistically significant reduction in cell viability. In the 48-h period, the reduction was considered statistically significant at concentrations of 200 and 400 µg/mL, and within 72 h, at concentrations of 100, 200 and 400 µg/mL. In this study, the activity of these substances was also time–dose dependent (Table 3).

#### 3.1.3. In Vitro Cytotoxicity Studies on Colorectal Cancer Cell Lines

In 2022, the effect of DHA on the viability of human colorectal cancer cells (LS174T) was showed. The cells were cultured with DHA at concentrations of 50, 100 and 150 µM for 24, 48 and 72 h, and the MTT assay was performed for analysis (Table 4) [27].

The results showed a dose-dependent reduction in the viability of LS174T cells treated with DHA. At 100 and 150 µM, the viability reduction was significant at 24, 48 and 72 h of treatment. With the use of a concentration of 50 µM, the reduction in cell viability of LS174T was significant at 48 and 72 h of treatment (Table 4) [27].

In another study, a comparative analysis was performed between the effects of DHA and other fatty acids in the treatment of human colorectal cancer cells (WiDr and COLO 205) [35]. The treatments used were 125 µM DHA, EPA and linoleic acid (LA) for a period of 72 h. There was no reduction in cell viability considered statistically significant in the treatment with LA. A statistically significant reduction in cell viability was observed in treatments with EPA and DHA (Table 4).

In HCT-116 human colorectal cancer cell lines, DHA, punicic acid (PunA) and the two substances in association were tested using the PrestoBlue Reagent cell viability assay [20]. PunA is a lipid available in pomegranate seed oil, identified as a conjugated isomer of alpha-linolenic acid, which is a compound of the omega-3 fatty acid family, and its potentially anticancer action has already been reported [39]. HCT-116 cells were treated with 100 µM DHA, 7 µM PunA and the combination of DHA plus PunA in a 1:1 ratio for a treatment period of 72 h [20] (Table 4). The results demonstrated a statistically significant reduction in cell viability in all tested treatments, as shown in Table 4 [20].

In another recent study, which is not shown in the tables, the effect of DHA in the absence or presence of isoliquiritigenin (ISL) on HCT-116 cells through the MTT assay after 24 h of treatment was analyzed [26]. In one of the analyses, the cells were treated with 20, 40, 60 and 80 µM DHA alone, and in another, the concentration of 20 µM DHA was associated with 10, 20 and 40 µM ISL. In the experiment where DHA was tested alone, its cytotoxic activity was dose dependent, and at concentrations of 80 µM and 160 µM, the cytotoxicity changed from weak to strong, respectively. The effect of ISL alone and associated with DHA also demonstrated dose-dependent cytotoxicity.

#### 3.1.4. In Vitro Cytotoxicity Studies on Prostate Cancer Cell Lines

Table 5 presents three in vitro cytotoxicity studies performed using MTT assays on prostate cancer cell lines. It shows the comparative analysis between the DHA and EPA fatty acids and omega-6 arachidonic acid (AA), in which DU-145 cells were treated with concentrations of 10, 25, 50 and 100 µM for a 24-h period. These same authors carried out studies comparing treatment periods of 12, 24, 48 and 72 h of DU-145 cells with DHA alone (Table 5) [44]. In another study, also provided in Table 5, DHA (25, 50, 100 and 150 µM) and the anticancer drug docetaxel (0.1, 0.5, 1 and 4 µM) in prostate cancer cell lines (PC3) and drug-resistant prostate cancer cells (PC3R) for 24 h and 48 h was analyzed [19].

The results of the comparative study between fatty acids demonstrated that DHA caused a statistically significant cytotoxic effect at concentrations of 10, 25, 50 and 100 µM; EPA, at concentrations of 25, 50 and 100 µM; and AA showed no statistically significant reduction in DU145 cells (Table 5). In a comparative analysis with the time variable, it was reported that the concentration of 50 µM was cytotoxic with a *p* value < 0.05 at all times tested (Table 5) [44].

In another in vitro analysis, within 24 h, docetaxel caused cell inhibition at all concentrations tested (0.1, 0.5, 1 and 4 µM) in the PC3 cell line; however, docetaxel only demonstrated inhibitory activity against PC3R cells at a concentration of 0.5 µM [19]. When observing the activity of DHA alone under the same conditions for time and cell lines, there was a significant reduction in PC3R at concentrations of 100 and 150 µM and in PC3 at concentrations of 50, 100 and 150 µM. The 25 µM concentration of isolated DHA did not demonstrate statistically significant activity against prostate cancer in the PC3 and PCR3 cell lines (Table 5) [19]. However, when incubating DHA alone and docetaxel alone for a period of 48 h, docetaxel significantly inhibited both lineages at all tested concentrations (0.1, 0.5, 1 and 4 µM). In this study, in the 48-h incubation time, isolated DHA also significantly inhibited both lineages at concentrations from 50 to 150 µM. At this time, the 25 µM concentration also did not show a statistically significant result for the cell inhibition of both lineages (Table 5) [19].

After these tests, a comparative analysis of DHA alone and in association with the substances LY294002 (PI3K inhibitor), MK2206 (AKT inhibitor) and Ferrostatin1 (ferroptosis inhibitor) was also performed (Table 5). PI3K/AKT are protein signaling pathways that maintain cell proliferation and drug resistance [19]. Ferroptosis is among the mechanisms that causes cell death [19]. DHA in the presence of LY294002 and MK2206 reduced PC3R cell viability, and in the presence of ferrostatin1, it did not show the ability to reduce PC3R cell viability (Table 5).

In another analysis, which is not shown in the tables, it was demonstrated through the MTT assay that combinations of docetaxel (0.1 µM) plus DHA (25 µM or 50 µM) caused a greater reduction in the cell viability of prostate cancer than that obtained when the isolated substances were applied at 24 h and 48 h of exposure [19]. DHA has been tested alone and in combination with lipopolysaccharide (LPS), a substance that induces inflammation [46], which is a process that promotes carcinogenesis [1]. Cell inhibition only occurred when DHA alone was applied to the PC3 cell line at a concentration of 100 µM (Table 5).

#### 3.1.5. In Vitro Cytotoxicity Studies on Stomach Cell Lines

Tumor (AGS) and nontumor cell lines of the stomach (GES-1) were subjected to treatment with DHA and cisplatin (Cisp), which is an antineoplastic agent, for a period of 48 h, and then the MTT test was performed [18] (Table 6).

The results found indicated that DHA was cytotoxic for AGS and noncytotoxic for GES-1, while CisP was toxic for the two cell lines tested (Table 6) [18]. In another study, which is not shown in the tables, AGS cells were treated with DHA (7.5; 15; 22.5; 30; 37.5; 45 µg/mL), 5-fluorouracil (1.5625; 3.125; 6, 25; 12.5; 25; 50 µg/mL) and DHA in association with 5-fluorouracil (5-FU) at a 1:1 ratio at all concentrations for a period of 24 h and 48 h of treatment, and the evaluation of cellular inhibition was performed through the MTT assay [32]. Within 24 h of treatment, DHA caused cytotoxicity in AGS cells at a concentration of 30 µg/mL, 5-FU at 12.5 µg/mL and the combination of DHA plus 5-FU at 15 µg/mL + 3.125 µg/mL. Within 48 h, the cytotoxic effect was observed from 15 µg/mL in the treatment with DHA alone, 3.125 µg/mL with 5-FU alone and 7.5 µg/mL plus 1.5625 µg/mL with the association of DHA + 5-FU. Cell inhibition activity was increased in a time–dose-dependent manner in all treatments [32].

#### 3.1.6. In Vitro Cytotoxicity Studies on Liver Cell Lines

Liver tumor and nontumor cell lines were subjected to comparative analyses using DHA alone, other omega 3, omega 6 and omega 9 fatty acids, other drugs, including antineoplastic agents and substances synthesized from DHA [41], as shown in Table 7.

Cell lines of human hepatic carcinoma (HepG2), normal human hepatocytes (HP-F) and rat hepatocytes (RTCP10) were exposed to concentrations of 1, 10, 50 and 100 µM DHA, EPA, monounsaturated fatty acid omega-9 oleic acid (OA), omega-6 linoleic acid (LA), omega-3 α-linolenic acid (ALA), PZ-DHA ester, PZ-EPA ester, oleic acid ester of Pz (PZ-OA ester), linoleic acid ester of Pz (PZ-LA ester), α-linolenic acid ester of Pz (PZ-ALA-ester), phloridzin (PZ), PZ-stearic acid ester, stearic acid, sorafenib and phoretin for a period of 24 h [41]. The substances that caused inhibition only for liver cancer cell lines were DHA (100 µM), EPA (100 µM), PZ-DHA ester (50, 100 µM), PZ-EPA ester (50, 100 µM) and PZ-ALA ester (50 and 100 µM) [41]. The substances that showed inhibitory activity in the three lineages were PZ-OA ester, PZ-LA ester and PZ-stearic acid ester. Chemical compounds that did not show inhibitory activity in any cell line studied by Nair, Ziaullah and Rupasinghe (2014) [41] were OA, LA, ALA, PZ and stearic acid. Two substances, sorafenib and phoretin, only inhibited HepG2 and RTCP10 cells. The 100 µM concentration was present in all results that showed statistically significant inhibitions of cell lines, and the 1 µM concentration did not cause cell inhibition in any cell lineage.

### 3.2. Human Studies

The effects of omega-3 supplementation in the diet of colorectal cancer patients were studied. A randomized trial with 11 patients with colorectal cancer was conducted. For 9 weeks, a group of 6 patients, called Group A, ingested 2 g/day of fish oil (approximately 600 mg/day of EPA and DHA acid), and the second group with 5 patients, Group B, was the untreated group. Both groups were evaluated one day before the first chemotherapy session and after 9 weeks of treatment. Group A showed, after the study period, an increase in EPA and DHA in blood plasma of 1.8 and 1.4 times, respectively, muscle mass gain (mean of +1.2 kg), a greater tolerance to chemotherapy and a decrease in the tumor, while the untreated group showed loss of muscle mass (mean −0.5 kg), lower tolerance to chemotherapy and no significant increase in EPA and DHA plasma [40].

Another study was conducted with 128 patients who had some types of gastrointestinal cancer (including gastric and colorectal) and cachexia. These patients were provided a diet with or without 1.1 g of EPA, 0.5 g of DHA and 16 g of protein. Chronologically, the biochemical and physiological state of these patients was observed using bioelectrical impedance analysis. The authors concluded that the fish oil-enriched diet helped both with chemotherapy tolerance and with tumor shrinkage and increased lean body mass over time [43].

Another study was conducted to determine the maximum dose and toxicity of capsules containing *n*-3 fatty acids [28]. These authors reported that it is important to use the maximum tolerated dose in an attempt to reverse or minimize the cachexia presented by patients. They also reported that patients with advanced cancer tolerated a high dose of GA *n*-3 in capsules, with few side effects (mainly diarrhea), and suggested an appropriate dose of 0.3 g/kg per day for 17 days. In a second phase of the study, the authors reported stabilization or weight gain in only a minority of patients, but they considered that the *n*-3 GAs are still useful as adjuvant therapy in the treatment of cancer patients who have incurred significant weight loss [28]. In breast cancer and other types of cancer, at a minimum dose of 2.0 g/day of EPA + DHA, it was possible to verify tissue enrichment in humans of these fatty acids, similar to studies with animals. This dose can be used for conducting clinical trials [30].

## 4. Discussion

DHA is present in several scientific articles in the field of cancer therapy, in which they report the efficiency of this bioactive compound against different types of malignant neoplasms, including those listed in the list of highest incidence and mortality. Anticancer activity was observed in studies involving cell lines that were treated with DHA alone, combined with other substances, including antineoplastics, and when molecules derived from DHA were used.

One recent study on the in vitro anticancer activity of DHA was in the field of nanomedicine. In vitro experiments with DHA were associated with TS and DOX drugs transported in nanostructured lipids (NLC-DHA-TS-DOX), resulting in strongly cytotoxic cell inhibition against breast cancer (4T1 cell line) [37]. In this study, the NLC-DHA nanoparticles did not show cytotoxic activity at the tested concentration. In this 4T1 lineage, DHA was also tested [38] with DHA at concentrations of 10, 20, 30, 40 and 50 µM, and the result also did not cause cytotoxicity. This cell line was also treated with PZ and PZ-DHA, and there was no cytotoxic effect [38]. Cytotoxicity results were obtained with this cell line at concentrations of 50, 100 and 150 µM DHA at 24 h and 48 h, but at concentrations of 10 and 25 µM, there was no cytotoxic effect [48]. However, they did not observe a statistically significant result of cell inhibition at concentrations of 10, 20 and 30 µM within 72 h of treatment with DHA [31]. The studies carried out by these authors differed in terms of the type of in vitro cell viability analysis assay, treatment time and, in some cases, the concentration used.

The SUM-149 cell line was tested with DHA, PZ and PZ-DHA [31]. Statistically significant cytotoxic activity was observed after treatment with DHA and PZ-DHA at concentrations of 30, 40 and 50 µM. This cytotoxic effect was not observed by the same authors when SUM-149 cells were treated with PZ. This result demonstrates that the type of drug tested interferes with the cell inhibition effect [31]. This can also be observed on MDA-MB-231 cells within 24 h with the drug 13R,20-diHDHA, and the study did not obtain statistically significant results at any of the tested concentrations [45]. A noncytotoxic result was also observed regarding the 4-OXO-DHA molecule [42].

Studies with other DHA-derived molecules were also performed [42]. The substance 4-OH-DHA was analyzed within 96 h at a concentration of 100 µM and showed cytotoxicity in the cell lines MCF-10F, trMCF, bsMCF, MDA-MB-231 and SK-BR-3, with the exception of cytotoxic activity in the tested cell line T-47D. When the same authors tested the substance 4-OXO-DHA, they obtained a statistically significant cytotoxicity result in all these lineages [42]. The PZ-DHA ester substance was toxic to liver cancer but not to the HP-F and RTCP10 lineages [41].

The concentration of 100 µM DHA demonstrated a cytotoxic effect in breast tumor and nontumor cell lines. This concentration was also toxic to lung [17], colorectal [20,37], prostate [19], stomach [18] and liver cell lines [41]. However, in the normal cell lines of the stomach (GES-1) [18] and liver (HP-F and RTCP10) [41], there was no statistically significant cytotoxic activity.

Cytotoxic analysis of DHA was tested comparatively with other fatty acids. In comparison with omega 3 EPA, the cytotoxic effect was shown by both substances in colorectal [35], prostate [44] and liver [41] cancer cell lines. In comparison with omega 3 (ALA), in the liver cancer cell line, only DHA was cytotoxic [41]. In comparison with omega 6 (LA), both in the colorectal cancer cell line and in the liver cancer cell line, only DHA showed cytotoxic activity that was considered statistically significant. In comparison with omega 6 (AA), in the prostate cancer cell lines, only DH was cytotoxic. In comparison with omega 9 (OA), in the liver cancer cell line, only DHA was cytotoxic. These analyses corroborate with other information, which mentioned that the disposition and quantity of unsaturation in DHA interferes with the biological activity of fatty acids [14,15].

In other studies, the cytotoxic activity of DHA alone or associated with other substances was observed. A comparison was made between DHA alone, apatinib alone and the two substances in combination [38]. The results showed that the combination of the two substances showed a synergistic effect against breast cancer. The synergistic effect was also observed in the association of nanocarried DHA with TS and DOXO in breast cancer [37], in the association of DHA with PunA in colorectal cancer [20] and with ISL in colorectal cancer [26].

The administration of DHA in cancer patients has been shown to be a coadjuvant in chemotherapy treatment. The addition of DHA in the diet, either in supplementation associated with EPA and/or proteins, helped in the process of muscle mass gain, weight maintenance, tolerance to chemotherapy and tumor shrinkage [30,40,43]. The intake of 6 g of protein, 1.1 g of EPA and 0.5 g of DHA per day is recommended to increase lean mass gain [43], and the minimum dose of 2.0 g/day of EPA + DHA can be used in clinical trials and is sufficient for tissue enrichment to occur [30].

Thus, DHA exhibits cytotoxic action against different tumor lineages and can be ingested in the diet as an adjunct to cancer treatment, within the specific concentrations presented for each type of cancer. In children, these dosages may vary to lower dosage; however, further research on the use of this drug is still needed to standardize the protocol for use in different cancer lineages.

## 5. Final Considerations

The results obtained reveal the possibility of using DHA in therapies against cancer cell lines. In the scientific articles explored, most of the concentrations used with effectiveness varied between 10 µM and 200 µM DHA, which may support the choice of concentrations for future in vitro analyses. In addition, the results of the different articles analyzed showed that DHA, in association with other drugs, generated increased activity against cancer, which is considered a synergistic effect. Furthermore, DHA and its associated substances, as well as substances derived from DHA, generated different anticancer actions, according to the type of cell line studied. In relation to nonneoplastic cells, it did not present cytotoxicity or side effects in some studies.

Other therapeutic strategies that can still be analyzed are studies related to the activity of DHA in other types of cancer, as well as the association of this substance with medicinal plants.

This work contributes to the community by demonstrating the possible applicability of DHA in the pharmaceutical area of oncological therapies.

## Figures and Tables

**Table 1:** Articles included in this review with their respective first authors, journal and impact factor.

Author (Reference)	Journal	Impact Factor
Ahangar et al., 2016 [27]	*Journal of Cancer Research and Therapeutics*	1331
Aslan et al., 2020 [12]	*Life Sciences*	6.78
Bae et al., 2020 [13]	*Animal Cells Systems (Seoul)*	2.34
Bai et al., 2019 [17]	*Journal of Experimental and Clinical Cancer Research*	12.66
Bilici, 2014 [7]	*World Journal of Gastroenterology*	5742
Bray et al., 2018 [4]	*Cancer J Clin*	508.7
Burns et al., 1999 [28]	*Clin Cancer Research*	13.8
Dierge et al., 2021 [14]	*Cell Metabolism*	31.37
El-Ashmawy et al., 2020 [29]	*Life Sciences*	6.78
Fasano et al., 2017 [30]	*Food Science and Nutrition*	3483
Fernando et al., 2019 [31]	*Cancer Letters*	9756
Fu et al., 2018 [9]	*Frontiers in Pharmacology*	5988
Gao et al., 2016 [32]	*World Journal of Gastroenterology*	5374
Ghasemifard et al., 2015 [22]	*British Journal of Nutrition*	3334
Hanahan; Weinberg, 2011 [33]	*Cell*	66.85
Jiao et al., 2018 [34]	*BMC Cancer*	4.4
Jin et al., 2021 [26]	*Molecular Biology Reports*	2742
Kato; Kolenic; Pardini, 2007 [35]	*Nutrition and Cancer*	2322
Khalid et al., 2022 [21]	*International Journal of Food Properties*	3338
Kim et al., 2015 [36]	*Hindawi*	3915
Lages et al., 2020 [37]	*Biomedicine and Pharmacotherapy*	7419
Li et al., 2021 [6]	*World Journal of Gastroenterology*	5742
LV; Xu, 2022 [23]	*A Review. Foods*.	6.043
Ma et al., 2020 [38]	*Bioscience, Biotechnology and Biochemistry*	1063
Melo et al., 2014 [39]	*Journal’s Impact IF of Nutrition and Food Science*	1.37
Mocellin et al., 2013 [40]	*Lipids*	1646
Nair; Ziaullah; Vasantha Rupasinghe, 2014 [41]	*PLoS ONE*	3752
Nurgali; Jagoe; Abalo, 2018 [10]	*Front Pharmacol*	5988
Ortega et al., 2021 [18]	*European Journal of Pharmacology*	4432
Park; Lim; Kim, 2018 [25]	*Nutrients*	6706
Patterson et al., 2012 [15]	*Journal of Nutrition and Metabolism*	2.79
Pizato et al., 2018 [11]	*Scientific Reports*	4996
Pogash et al., 2015 [42]	*In Vitro Cellular and Developmental Biology—Animal*	2723
Shao et al., 2022 [19]	*Folia Biologica (Praha)*	1.182
Shirai et al., 2017 [43]	*Scientific Reports*	4996
Sitarz et al., 2018 [8]	*Frontiers in Pharmacology*	5988
Sun et al., 2017 [44]	*Lipids in Health and Disease*	4.15
Sung et al., 2021 [5]	*Cancer J Clin*	508.7
Tasaki et al., 2017 [16]	*Experimental and Therapeutic Medicine*	2751
Vermonden et al., 2021 [20]	*Nutrients*	6706
Wang et al., 2021 [45]	*Antioxidants*	7675
Wood; Harper, 2021 [24]	*The Journal of Pediatrics*	4406
Wu et al., 2019 [46]	*Pharmazie*	1515
Yue et al., 2022 [47]	*Marine Drugs*	6085
Zhang et al., 2021 [48]	*Arabian Journal of Chemistry*	6212

**Table 2:** Analysis results of the in vitro cytotoxicity tests on breast nontumor and cancer cell line.

Reference	Analysis	t	Cell Line	Inhibition Cell					
				DHA [ ]	*p*-Value	Apatinib [ ]	*p*-Value		
Ma et al., 2020 [38]	Cell viability CCK-8 kit	48 h	MDA-MB-231 (human breast cancer)	0 µM	ns	0 µM	ns	-	
				25 µM	*	12.5 µM	*		
				50 µM	**	25 µM	**		
				100 µM	**	50 µM	***		
				200 µM	***	75 µM	***		
				400 µM	***	100 µM	***		
				600 µM	***	200 µM	***		
				800 µM	***				
				**DHA [ ]**	***p*-value**	**Apatinib [ ]**	***p*-value**	**DHA + Apatinib [ ]**	***p*-value**
				0 µM	ns	0 µM	ns	0 µM	ns
				75 µM	*	40.5 µM	*	75 µM + 40.5 µM	***
				112.5 µM	*	40.5 µM	*	112.5 µM + 40.5 µM	***
				150 µM	**	54 µM	**	150 µM + 54 µM	***
Fernando et al., 2019 [31]	Cell viability 7-AAD staining	72 h	MDA-MB-231 (human breast cancer)	**DHA [ ]**	***p*-value**	**PZ [ ]**	***p*-value**	**PZ-DHA[ ]**	***p*-value**
				0 µM	ns	0 µM	ns	0 µM	ns
				10 µM	ns	10 µM	ns	10 µM	ns
				20 µM	ns	20 µM	ns	20 µM	ns
				30 µM	ns	30 µM	ns	30 µM	ns
				40 µM	*	40 µM	ns	40 µM	*
				50 µM	*	50 µM	ns	50 µM	*
			SUM-149 (human breast cancer)	0 µM	ns	0 µM	ns	0 µM	ns
				10 µM	ns	10 µM	ns	10 µM	ns
				20 µM	ns	20 µM	ns	20 µM	ns
				30 µM	***	30 µM	ns	30 µM	***
				40 µM	***	40 µM	ns	40 µM	***
				50 µM	***	50 µM	ns	50 µM	***
			4T1 (mouse breast cancer)	0 µM	ns	0 µM	ns	0 µM	ns
				10 µM	ns	10 µM	ns	10 µM	ns
				20 µM	ns	20 µM	ns	20 µM	ns
				30 µM	ns	30 µM	ns	30 µM	ns
				40 µM	ns	40 µM	ns	40 µM	ns
				50 µM	ns	50 µM	ns	50 µM	ns
Pogash et al., 2015 [42]	Cell viability MTT assay	96 h	MCF-10F (normal-like breast cell)	**DHA [ ]**	***p*-value**	**4-OH-DHA [ ]**	***p*-value**	**4-OXO-DHA [ ]**	***p*-value**
				0 µM	ns	0 µM	ns	0 µM	ns
				100 µM	*	100 µM	*	100 µM	*
			trMCF (transformed breast cell)	0 µM	ns	0 µM	ns	0 µM	ns
				100 µM	*	100 µM	*	100 µM	*
			bsMCF (basal breast cancer)	0 µM	ns	0 µM	ns	0 µM	ns
				100 µM	*	100 µM	*	100 µM	*
			MDA-MB-231 (basal breast cancer)	0 µM	ns	0 µM	ns	0 µM	ns
				100 µM	*	100 µM	*	100 µM	*
			T-47D (luminal breast cancer)	0 µM	ns	0 µM	ns	0 µM	ns
				100 µM	*	100 µM	ns	100 µM	*
			SK-BR-3 (luminal breast cancer)	0 µM	ns	0 µM	ns	0 µM	ns
				100 µM	*	100 µM	*	100 µM	*
			MDA-MB-231 (basal breast cancer)	0 µM	ns	0 µM	ns	0 µM	ns
				10 µM	ns	10 µM	*	10 µM	ns
				25 µM	*	25 µM	*	25 µM	ns
				50 µM	*	50 µM	*	50 µM	*
				100 µM	*	100 µM	*	100 µM	*
		24 h	MDA-MB-231 (basal breast cancer)	**4-OXO-DHA [ ]**	***p*-value**				
				0 µM	ns	-		-	
				10 µM	ns				
				25 µM	ns				
				50 µM	ns				
				100 µM	ns				
		48 h	MDA-MB-231 (basal breast cancer)	0 µM	ns	-		-	
				10 µM	ns				
				25 µM	ns				
				50 µM	ns				
				100 µM	*				
		72 h	MDA-MB-231 (basal breast cancer)	0 µM	ns	-		-	
				10 µM	ns				
				25 µM	ns				
				50 µM	*				
				100 µM	*				
		96 h	MDA-MB-231 (basal breast cancer)	0 µM	ns	-		-	
				10 µM	ns				
				25 µM	ns				
				50 µM	*				
				100 µM	*				
Wang et al., 2021 [45]	CellTiter 96^®^ AQueous One Solution kit	24 h	MDA-MB-231 (basal breast cancer)	**13R,20-diHDHA [ ]**	***p*-value**				
				0 µM	ns	-		-	
				1 µM	ns				
				10 µM	ns				
				20 µM	ns				
				30 µM	ns				
				40 µM	ns				
			MCF-10F (normal-like breast cell)	0 µM	ns	-		-	
				1 µM	ns				
				10 µM	ns				
				20 µM	ns				
				30 µM	ns				
				40 µM	ns				

DHA: docosahexaenoic acid; PZ: phloridizin; PZ-DHA: phloridizin docosahexanoate. 4-OH-DHA: 4-hydroxy-docosahexaenoic acid. 4-OXO-DHA: 4-oxo-docosahexaenoic. 13R,20-diHDHA: 13R,20-dihydroxydocosahexaenoic acid. [ ]: concentration; Cell Counting Kit-8 (CCK-8). 7-AAD: colorimetric reagent 7-aminoactinomycin. MTT (3-(4,5-dimethylthiazolyl-2)-2,5-diphenyltetrazolium bromide) assay. When there was a statistically significant difference, the results in the graphs are shown as follows: * *p* value < 0.05; ** *p* value < 0.01; *** *p* value < 0.001. The results with *p* values ≥ 0.05 were considered nonsignificant (ns: nonsignificant).

**Table 3:** Analysis results of the in vitro cytotoxicity tests on lung cancer cell lines.

Reference	Analysis	t	Cell Line	Inhibition Cell					
				DHA [ ]	*p*-Value				
Kim et al., 2015 [36]	Cell viability MTT assay	24 h	A549 (human lung cancer)	0 µM	ns	-		-	
				10 µM	ns				
				30 µM	***				
				60 µM	***				
			H1299 (human lung cancer)	0 µM	ns	-		-	
				10 µM	ns				
				30 µM	ns				
				60 µM	***				
				**DHA [ ]**	***p*-value**				
Bai et al., 2019 [17]	Cell viability WST assay	24 h	A549 (human lung cancer)	0 µM	ns	-		-	
				25 µM	ns				
				50 µM	ns				
				75 µM	**				
				100 µM	**				
			H1299 (human lung cancer)	0 µM	ns	-		-	
				25 µM	ns				
				50 µM	*				
				75 µM	**				
				100 µM	**				
			LLC (mouse lung cancer)	0 µM	ns	-		-	
				25 µM	ns				
				50 µM	**				
				75 µM	**				
				100 µM	**				
Yue et al., 2022 [47]	Cell viability MTT assay	24 h	95D (human lung cancer cells)	**DHA-PC [ ]**	***p*-value**	**DHA-TG [ ]**	***p*-value**	**DHA-EE [ ]**	***p*-value**
				0 µg/mL	ns	0 µg/mL	ns	0 µg/mL	ns
				50 µg/mL	*	50 µg/mL	ns	50 µg/mL	ns
				100 µg/mL	**	100 µg/mL	*	100 µg/mL	ns
				200 µg/mL	**	200 µg/mL	**	200 µg/mL	ns
				400 µg/mL	**	400 µg/mL	**	400 µg/mL	*
		48 h	95D (human lung cancer cells)	0 µg/mL	ns	0 µg/mL	ns	0 µg/mL	ns
				50 µg/mL	*	50 µg/mL	ns	50 µg/mL	ns
				100 µg/mL	**	100 µg/mL	**	100 µg/mL	ns
				200 µg/mL	**	200 µg/mL	**	200 µg/mL	*
				400 µg/mL	**	400 µg/mL	**	400 µg/mL	**
		72 h	95D (human lung cancer cells)	0 µg/mL	ns	0 µg/mL	ns	0 µg/mL	ns
				50 µg/mL	*	50 µg/mL	*	50 µg/mL	ns
				100 µg/mL	**	100 µg/mL	**	100 µg/mL	*
				200 µg/mL	**	200 µg/mL	**	200 µg/mL	*
				400 µg/mL	**	400 µg/mL	**	400 µg/mL	**

DHA: docosahexaenoic acid; DHA-PC: DHA-enriched phosphatidylcholine; DHA-TG: DHA-triglyceride; DHA-EE: DHA-ethyl esters; [ ]: concentration; MTT (3-(4,5-dimethylthiazolyl-2)-2,5-diphenyltetrazolium bromide) assay; WST: water-soluble tetrazolium. When there was a statistically significant difference, the results in the graphs are shown as follows: * *p* value < 0.05; ** *p* value < 0.01; *** *p* value < 0.001. The results with *p* values ≥ 0.05 were considered nonsignificant (ns: nonsignificant).

**Table 4:** Analysis results of the in vitro cytotoxicity tests on colorectal cancer cell lines.

Reference	Analysis	t	Cell Line	Inhibition Cell					
				DHA [ ]	*p*-Value				
Ahangar et al., 2016 [27]	Cell viability MTT assay	24 h	LS174T (human colorectal cancer)	0 µM	ns	-		-	-
				50 µM	ns				
				100 µM	***				
				150 µM	***				
		48 h	LS174T (human colorectal cancer)	0 µM	ns	-		-	-
				50 µM	***				
				100 µM	***				
				150 µM	***				
		72 h	LS174T (human colorectal cancer)	0 µM	ns	-		-	-
				50 µM	***				
				100 µM	***				
				150 µM	***				
Kato et al., 2007 [35]	BrdU incorporation assay	72 h		**DHA [ ]**	***p*-value**	**EPA [ ]**	***p*-value**	**LA [ ]**	***p*-value**
			WiDr (human colorectal carcinoma)	0 µM	ns	0 µM	ns	0 µM	ns
				125 µM	*	125 µM	*	125 µM	ns
			COLO 205 (human colorectal carcinoma)	0 µM	ns	0 µM	ns	0 µM	ns
				125 µM	*	125 µM	*	125 µM	ns
				**DHA [ ]**	***p*-value**	**PunA [ ]**	***p*-value**	**DHA + PunA [ ]**	***p*-value**
Vermonden et al., 2021 [20]	Cell viability PrestoBlue Reagent	72 h	HCT-116 (human colorectal cancer)	0 µM	ns	0 µM	ns	0 µM	ns
				100 µM	***	7 µM	***	100 µM + 7 µM	***

DHA: docosahexaenoic acid; EPA: eicosapentaenoic acid; LA: linoleic acid; PunA: punicic acid. [ ]: concentration; MTT (3-(4,5-dimethylthiazolyl-2)-2,5-diphenyltetrazolium bromide) assay. When there was a statistically significant difference, the results in the graphs are shown as follows: * *p* value < 0.05; *** *p* value < 0.001. The results with *p* values ≥ 0.05 were considered nonsignificant (ns: nonsignificant).

**Table 5:** Analysis results of the in vitro cytotoxicity tests on prostate cancer cell lines. DHA: docosahexaenoic acid; EPA: eicosapentaenoic acid.

Reference	Analysis	t	Cell Line	Inhibition Cell							
				DHA [ ]	*p*-Value	EPA [ ]	*p*-Value	AA [ ]	*p*-Value		
Sun et al., 2017 [44]	Cell viability MTT assay	24 h	DU145 (human prostate carcinoma)	0 µM	ns	0 µM	ns	0 µM	ns	-	
				10 µM	*	10 µM	ns	10 µM	ns		
				25 µM	*	25 µM	*	25 µM	ns		
				50 µM	*	50 µM	*	50 µM	ns		
				100 µM	*	100 µM	*	100 µM	ns		
		12 h	DU145 (human prostate carcinoma)	0 µM	ns	-		-		-	
				50 µM	*						
		24 h	DU145 (human prostate carcinoma)	0 µM	ns						
				50 µM	*						
		48 h	DU145 (human prostate carcinoma)	0 µM	ns						
				50 µM	*						
		72 h	DU145 (human prostate carcinoma)	0 µM	ns						
				50 µM	*						
Shao et al., 2022 [19]	Cell viability MTT assay			**DHA [ ]**	***p*** **-value**	**Docetaxel [ ]**	***p*** **-value**				
		24 h	PC3 (human prostate cancer line)	0 µM	ns	0 µM	ns	-		-	
				25 µM	ns	0.1 µM	*				
				50 µM	*	0.5 µM	*				
				100 µM	*	1 µM	*				
				150 µM	*	4 µM	*				
			PC3R (drug-resistant prostate cancer cell line)	0 µM	ns	0 µM	ns	-		-	
				25 µM	ns	0.1 µM	ns				
				50 µM	ns	0.5 µM	*				
				100 µM	*	1 µM	ns				
				150 µM	*	4 µM	ns				
		48 h	PC3 (human prostate cancer line)	0 µM	ns	0 µM	ns	-		-	
				25 µM	ns	0.1 µM	*				
				50 µM	*	0.5 µM	*				
				100 µM	*	1 µM	*				
				150 µM	*	4 µM	*				
			PC3R (drug-resistant prostate cancer cell line)	0 µM	ns	0 µM	ns	-		-	
				25 µM	ns	0.1 µM	*				
				50 µM	*	0.5 µM	*				
				100 µM	*	1 µM	*				
				150 µM	*	4 µM	*				
		24 h	PC3R (drug-resistant prostate cancer cell line)	**DHA [ ]**	***p*** **-value**	**DHA + LY294002 [ ]**	***p*** **-value**	**DHA + MK2206 [ ]**	***p*** **-value**	**DHA + Ferrostatin1 [ ]**	***p*** **-value**
				0 µM	ns	0 µM	ns	0 µM	ns	0 µM	ns
				100 µM	*	100 µM + 10 µM	*	100 µM + 0.1 µM	*	100 µM + 5 µM	ns
Wu et al., 2019 [46]	Cell viability MTT assay			**DHA [ ]**	***p*** **-value**	**DHA + LPS [ ]**	***p*** **-value**				
		24 h	PC3 (human prostate cancer line)	0 µM	ns	0 µM + 0 ng/mL	ns	-		-	
				25 µM	ns	25 µM + 10 ng/mL	ns				
				75 µM	ns	75 µM + 10 ng/mL	ns				
				100 µM	*	100 µM + 10 ng/mL	ns				

AA: arachidonic acid; LY294002: PI3K inhibitor; MK2206: AKT inhibitor; Ferrostatin1: ferroptosis inhibitor; [ ]: concentration; MTT (3-(4,5-dimethylthiazolyl-2)-2,5-diphenyltetrazolium bromide) assay. When there was a statistically significant difference, the results in the graphs are shown as follows: * *p* value < 0.05. The results with *p* values ≥ 0.05 were considered nonsignificant (ns: nonsignificant).

**Table 6:** Analysis results of the in vitro cytotoxicity tests on stomach nontumor (GES-1) and gastric cancer (AGS) cell lines.

Reference	Analysis	t	Cell Line	Inhibition Cell			
				DHA [ ]	*p* Value	CisP [ ]	*p* Value
				0 µM	ns	0 µM	ns
Ortega et al., 2021 [18]	Cell viability MTT assay	48 h	AGS (human gastric cancer)	25 µM	**	50 µM	**
				50 µM	**		
				100 µM	**		
				0 µM	ns	0 µM	ns
			GES-1 (human nontumoral)	25 µM	ns	50 µM	**
				50 µM	ns		
				100 µM	ns		

DHA: docosahexaenoic acid; [ ]: concentration; CisP: cisplatin; MTT (3-(4,5-dimethylthiazolyl-2)-2,5-diphenyltetrazolium bromide) assay. When there was a statistically significant difference, the results in the graphs are shown as follows: ** *p* value < 0.01. The results with *p* values ≥ 0.05 were considered nonsignificant (ns: nonsignificant).

**Table 7:** Analysis results of the in vitro cytotoxicity tests on liver nontumor and tumor cell lines.

Reference	Analysis	t	Cell Line	Inhibition Cell									
				DHA [ ]	*p* Value	EPA [ ]	*p* Value	OA [ ]	*p* Value	LA [ ]	*p* Value	ALA [ ]	*p* Value
Nair, Ziaullah and Rupasingher, 2014 [41]	Cell viability MTT assay	24 h		0 µM	ns	0 µM	ns	0 µM	ns	0 µM	ns	0 µM	ns
			HepG2 (human hepatic carcinoma)	1 µM	ns	1 µM	ns	1 µM	ns	1 µM	ns	1 µM	ns
				10 µM	ns	10 µM	ns	10 µM	ns	10 µM	ns	10 µM	ns
				50 µM	ns	50 µM	ns	50 µM	ns	50 µM	ns	50 µM	ns
				100 µM	*	100 µM	*	100 µM	ns	100 µM	ns	100 µM	ns
			HP-F (normal human hepatocytes)	0 µM	ns	0 µM	ns	0 µM	ns	0 µM	ns	0 µM	ns
				1 µM	ns	1 µM	ns	1 µM	ns	1 µM	ns	1 µM	ns
				10 µM	ns	10 µM	ns	10 µM	ns	10 µM	ns	10 µM	ns
				50 µM	ns	50 µM	ns	50 µM	ns	50 µM	ns	50 µM	ns
				100 µM	ns	100 µM	ns	100 µM	ns	100 µM	ns	100 µM	ns
			RTCP10 (rat hepatocytes)	0 µM	ns	0 µM	ns	0 µM	ns	0 µM	ns	0 µM	ns
				1 µM	ns	1 µM	ns	1 µM	ns	1 µM	ns	1 µM	ns
				10 µM	ns	10 µM	ns	10 µM	ns	10 µM	ns	10 µM	ns
				50 µM	ns	50 µM	ns	50 µM	ns	50 µM	ns	50 µM	ns
				100 µM	ns	100 µM	ns	100 µM	ns	100 µM	ns	100 µM	ns
				**PZ-DHA Ester [ ]**	***p* value**	**PZ-EPA Ester [ ]**	***p* value**	**PZ-OA Ester [ ]**	***p* value**	**PZ-LA Ester [ ]**	***p* value**	**PZ-ALA-Ester [ ]**	***p* value**
				0 µM	ns	0 µM	ns	0 µM	ns	0 µM	ns	0 µM	ns
			HepG2 (human hepatic carcinoma)	1 µM	ns	1 µM	ns	1 µM	ns	1 µM	ns	1 µM	ns
				10 µM	ns	10 µM	ns	10 µM	ns	10 µM	ns	10 µM	ns
				50 µM	*	50 µM	*	50 µM	*	50 µM	*	50 µM	*
				100 µM	*	100 µM	*	100 µM	*	100 µM	*	100 µM	*
			HP-F (normal human hepatocytes)	0 µM	ns	0 µM	ns	0 µM	ns	0 µM	ns	0 µM	ns
				1 µM	ns	1 µM	ns	1 µM	ns	1 µM	ns	1 µM	ns
				10 µM	ns	10 µM	ns	10 µM	ns	10 µM	ns	10 µM	ns
				50 µM	ns	50 µM	ns	50 µM	ns	50 µM	ns	50 µM	ns
				100 µM	ns	100 µM	ns	100 µM	*	100 µM	*	100 µM	ns
			RTCP10 (rat hepatocytes)	0 µM	ns	0 µM	ns	0 µM	ns	0 µM	ns	0 µM	ns
				1 µM	ns	1 µM	ns	1 µM	ns	1 µM	ns	1 µM	ns
				10 µM	ns	10 µM	ns	10 µM	ns	10 µM	ns	10 µM	ns
				50 µM	ns	50 µM	ns	50 µM	ns	50 µM	ns	50 µM	ns
				100 µM	ns	100 µM	ns	100 µM	*	100 µM	*	100 µM	ns
				**PZ [ ]**	***p* value**	**PZ-Stearic Acid Ester [ ]**	***p* value**	**Stearic Acid [ ]**	***p* value**	**Sorafenib [ ]**	***p* value**	**Phoretin [ ]**	***p* value**
				0 µM	ns	0 µM	ns	0 µM	ns	0 µM	ns	0 µM	ns
			HepG2 (human hepatic carcinoma)	1 µM	ns	1 µM	ns	1 µM	ns	1 µM	ns	1 µM	ns
				10 µM	ns	10 µM	ns	10 µM	ns	10 µM	*	10 µM	ns
				50 µM	ns	50 µM	*	50 µM	ns	50 µM	*	50 µM	ns
				100 µM	ns	100 µM	*	100 µM	ns	100 µM	*	100 µM	*
			HP-F (normal human hepatocytes)	0 µM	ns	0 µM	ns	0 µM	ns	0 µM	ns	0 µM	ns
				1 µM	ns	1 µM	ns	1 µM	ns	1 µM	ns	1 µM	ns
				10 µM	ns	10 µM	ns	10 µM	ns	10 µM	ns	10 µM	ns
				50 µM	ns	50 µM	ns	50 µM	ns	50 µM	ns	50 µM	ns
				100 µM	ns	100 µM	*	100 µM	ns	100 µM	ns	100 µM	ns
			RTCP10 (rat hepatocytes)	0 µM	ns	0 µM	ns	0 µM	ns	0 µM	ns	0 µM	ns
				1 µM	ns	1 µM	ns	1 µM	ns	1 µM	ns	1 µM	ns
				10 µM	ns	10 µM	ns	10 µM	ns	10 µM	ns	10 µM	ns
				50 µM	ns	50 µM	ns	50 µM	ns	50 µM	*	50 µM	ns
				100 µM	ns	100 µM	*	100 µM	ns	100 µM	*	100 µM	*

DHA: docosahexaenoic acid; [ ]: concentration; EPA: eicosapentaenoic acid; OA: oleic acid; LA: linoleic acid; ALA: α-linolenic acid; PZ: phloridzin; MTT (3-(4,5-dimethylthiazolyl-2)-2,5-diphenyltetrazolium bromide) assay. When there was a statistically significant difference, the results in the graphs are shown as follows: * *p* value < 0.05. The results with *p* values ≥ 0.05 were considered nonsignificant (ns: nonsignificant).

## Data Availability

Not applicable.

## References

[1] Wang J.-J., Lei K.-F., Han F. (2018). Tumor Microenvironment: Recent Advances in Various Cancer Treatments. Eur. Rev. Med. Pharmacol. Sci..

[2] Instituto Nacional de Câncer José Alencar Gomes da Silva (2017). Estimativa 2018: Incidência de Câncer No Brasil.

[3] Instituto Nacional de Câncer José Alencar Gomes da Silva (2019). Estimativa 2020: Incidência de Câncer No Brasil.

[4] Bray F., Ferlay J., Soerjomataram I., Siegel R.L., Torre L.A., Jemal A. (2018). Global Cancer Statistics 2018: GLOBOCAN Estimates of Incidence and Mortality Worldwide for 36 Cancers in 185 Countries. CA Cancer J. Clin..

[5] Sung H., Ferlay J., Siegel R.L., Laversanne M., Soerjomataram I., Jemal A., Bray F. (2021). Global Cancer Statistics 2020: GLOBOCAN Estimates of Incidence and Mortality Worldwide for 36 Cancers in 185 Countries. CA Cancer J. Clin..

[6] Li K., Zhang A., Li X., Zhang H., Zhao L. (2021). Advances in clinical immunotherapy for gastric cancer. Biochim. Biophys. Acta BBA Rev. Cancer.

[7] Bilici A. (2014). Treatment options in patients with metastatic gastric cancer: Current status and future perspectives. World J. Gastroenterol..

[8] Sitarz R., Skierucha M., Mielko J., Offerhaus J., Maciejewski R., Polkowski W. (2018). Gastric cancer: Epidemiology, prevention, classification, and treatment. Cancer Manag. Res..

[9] Fu B., Wang N., Tan H.-Y., Li S., Cheung F., Feng Y. (2018). Multi-Component Herbal Products in the Prevention and Treatment of Chemotherapy-Associated Toxicity and Side Effects: A Review on Experimental and Clinical Evidences. Front. Pharmacol..

[10] Nurgali K., Jagoe R.T., Abalo R. (2018). Editorial: Adverse Effects of Cancer Chemotherapy: Anything New to Improve Tolerance and Reduce Sequelae?. Front. Pharmacol..

[11] Pizato N., Luzete B.C., Kiffer L.F.M.V., Corrêa L.H., De Oliveira Santos I., Assumpção J.A.F., Ito M.K., Magalhães K.G. (2018). Omega-3 docosahexaenoic acid induces pyroptosis cell death in triple-negative breast cancer cells. Sci. Rep..

[12] Aslan C., Maralbashi S., Kahroba H., Asadi M., Soltani-Zangbar M.S., Javadian M., Shanehbandi D., Baradaran B., Darabi M., Kazemi T. (2020). Docosahexaenoic acid (DHA) inhibits pro-angiogenic effects of breast cancer cells via down-regulating cellular and exosomal expression of angiogenic genes and microRNAs. Life Sci..

[13] Bae S., Kim M.-K., Kim H.S., Moon Y.-A. (2020). Arachidonic acid induces ER stress and apoptosis in HT-29 human colon cancer cells. Anim. Cells Syst..

[14] Dierge E., Debock E., Guilbaud C., Corbet C., Mignolet E., Mignard L., Bastien E., Dessy C., Larondelle Y., Feron O. (2021). Peroxidation of *n*-3 and *n*-6 polyunsaturated fatty acids in the acidic tumor environment leads to ferroptosis-mediated anticancer effects. Cell Metab..

[15] Patterson E., Wall R., Fitzgerald G.F., Ross R., Stanton C. (2012). Health Implications of High Dietary Omega-6 Polyunsaturated Fatty Acids. J. Nutr. Metab..

[16] Tasaki S., Horiguchi A., Asano T., Ito K., Asano T., Asakura H. (2017). Docosahexaenoic acid inhibits the phosphorylation of STAT3 and the growth and invasion of renal cancer cells. Exp. Ther. Med..

[17] Bai X., Shao J., Zhou S., Zhao Z., Li F., Xiang R., Zhao A.Z., Pan J. (2019). Inhibition of lung cancer growth and metastasis by DHA and its metabolite, RvD1, through miR-138-5p/FOXC1 pathway. J. Exp. Clin. Cancer Res..

[18] Ortega L., Lobos-González L., Reyna-Jeldes M., Cerda D., De la Fuente-Ortega E., Castro P., Bernal G., Coddou C. (2021). The Ω-3 fatty acid docosahexaenoic acid selectively induces apoptosis in tumor-derived cells and suppress tumor growth in gastric cancer. Eur. J. Pharmacol..

[19] Shao Z.C., Zhu B.H., Huang A.F., Su M.Q., An L.J., Wu Z.P., Jiang Y.J., Guo H., Han X.-Q., Liu C.-M. (2022). Original Article Docosahexaenoic Acid Reverses Epithelial-Mesenchymal Transition and Drug Resistance by Impairing the PI3K/AKT/ Nrf2/GPX4 Signalling Pathway in Docetaxel-Resistant PC3 Prostate Cancer Cells (docosahexaenoic acid/drug resistance/ferroptosis/GPX4/autophagy/prostate). Folia Biol..

[20] Vermonden P., Vancoppenolle M., Dierge E., Mignolet E., Cuvelier G., Knoops B., Page M., Debier C., Feron O., Larondelle Y. (2021). Punicic Acid Triggers Ferroptotic Cell Death in Carcinoma Cells. Nutrients.

[21] Khalid W., Gill P., Arshad M.S., Ali A., Ranjha M.M.A.N., Mukhtar S., Afzal F., Maqbool Z. (2022). Functional behavior of DHA and EPA in the formation of babies brain at different stages of age, and protect from different brain-related diseases. Int. J. Food Prop..

[22] Ghasemifard S., Hermon K., Turchini G.M., Sinclair A.J. (2015). Metabolic fate (absorption,*β*-oxidation and deposition) of long-chain*n*-3 fatty acids is affected by sex and by the oil source (krill oil or fish oil) in the rat. Br. J. Nutr..

[23] Lv W., Xu D. (2022). Docosahexaenoic Acid Delivery Systems, Bioavailability, Functionality, and Applications: A Review. Foods.

[24] Wood E.H., Harper C.A. (2021). Lipid supplement reduced ROP in premature infants. J. Pediatr..

[25] Park M., Lim J.W., Kim H. (2018). Docoxahexaenoic Acid Induces Apoptosis of Pancreatic Cancer Cells by Suppressing Activation of STAT3 and NF-κB. Nutrients.

[26] Jin H., Kim H.S., Yu S.T., Shin S.R., Lee S.H., Seo G.S. (2021). Synergistic anticancer effect of docosahexaenoic acid and isoliquiritigenin on human colorectal cancer cells through ROS-mediated regulation of the JNK and cytochrome c release. Mol. Biol. Rep..

[27] Ahangar P., Sam M.R., Nejati V., Habibian R. (2016). Treatment of undifferentiated colorectal cancer cells with fish-oil derived docosahexaenoic acid triggers caspase-3 activation and apoptosis. J. Cancer Res. Ther..

[28] Burns C.P., Halab S., Clamon G.H., Hars V., Wagner B.A., Hohl R.J., Lester E., Kirshner J.J., Vinciguerra V., Paskett E. (1999). Phase I Clinical Study of Fish Oil Fatty Acid Capsules forPatients with Cancer Cachexia: Cancer and Leukemia GroupB Study 9473. Clin. Cancer Res..

[29] El-Ashmawy N.E., Al-Ashmawy G.M., Amr E.A., Khedr E.G. (2020). Inhibition of lovastatin- and docosahexaenoic acid-initiated autophagy in triple negative breast cancer reverted resistance and enhanced cytotoxicity. Life Sci..

[30] Fasano E., Serini S., Cittadini A., Calviello G. (2017). Long-chain *n*-3 PUFA against breast and prostate cancer: Which are the appropriate doses for intervention studies in animals and humans?. Crit. Rev. Food Sci. Nutr..

[31] Fernando W., Coyle K., Marcato P., Rupasinghe H.V., Hoskin D.W. (2019). Phloridzin docosahexaenoate, a novel fatty acid ester of a plant polyphenol, inhibits mammary carcinoma cell metastasis. Cancer Lett..

[32] Gao K., Liang Q., Zhao Z.-H., Li Y.-F., Wang S.-F. (2016). Synergistic anticancer properties of docosahexaenoic acid and 5-fluorouracil through interference with energy metabolism and cell cycle arrest in human gastric cancer cell line AGS cells. World J. Gastroenterol..

[33] Hanahan D., Weinberg R.A. (2011). Hallmarks of cancer: The next generation. Cell.

[34] Jiao Y., Watts T., Xue J., Hannafon B., Ding W.-Q. (2018). Sorafenib and Docosahexaenoic Acid Act in Synergy to Suppress Cancer Cell Viability: A Role of Heme Oxygenase 1. BMC Cancer.

[35] Kato T., Kolenic N., Pardini R.S. (2007). Docosahexaenoic Acid (DHA), a Primary Tumor Suppressive Omega-3 Fatty Acid, Inhibits Growth of Colorectal Cancer Independent of p53 Mutational Status. Nutr. Cancer.

[36] Kim N., Jeong S., Jing K., Shin S., Kim S., Heo J.Y., Kweon G.-R., Park S.-K., Wu T., Park J.-I. (2015). Docosahexaenoic Acid Induces Cell Death in Human Non-Small Cell Lung Cancer Cells by Repressing mTOR via AMPK Activation and PI3K/Akt Inhibition. BioMed Res. Int..

[37] Lages E.B., Fernandes R.S., Silva J.D.O., de Souza M., Cassali G.D., de Barros A.L.B., Ferreira L.A.M. (2020). Co-delivery of doxorubicin, docosahexaenoic acid, and α-tocopherol succinate by nanostructured lipid carriers has a synergistic effect to enhance antitumor activity and reduce toxicity. Biomed. Pharmacother..

[38] Ma Y., Yu J., Li Q., Su Q., Cao B. (2020). Addition of docosahexaenoic acid synergistically enhances the efficacy of apatinib for triple-negative breast cancer therapy. Biosci. Biotechnol. Biochem..

[39] Melo I.L.P., de Teixeira E.B., Marin- Huachaca N. (2014). Pomegranate Seed Oil (*Punica Granatum* L.): A Source of Punicic Acid (Conjugated α-Linolenic Acid). J. Hum. Nutr. Food Sci..

[40] Mocellin M.C., Silva J.D.A.P.E., Camargo C., Fabre M.E.D.S., Gevaerd S., Naliwaiko K., Moreno Y.M.F., Nunes E., Trindade E.B.S.D.M. (2013). Fish Oil Decreases *C*-Reactive Protein/Albumin Ratio Improving Nutritional Prognosis and Plasma Fatty Acid Profile in Colorectal Cancer Patients. Lipids.

[41] Nair S.V.G., Ziaullah, Rupasinghe H.P.V. (2014). Fatty Acid Esters of Phloridzin Induce Apoptosis of Human Liver Cancer Cells through Altered Gene Expression. PLoS ONE.

[42] Pogash T.J., El-Bayoumy K., Amin S., Gowda K., De Cicco R.L., Barton M., Su Y., Russo I.H., Himmelberger J.A., Slifker M. (2019). Oxidized derivative of docosahexaenoic acid preferentially inhibit cell proliferation in triple negative over luminal breast cancer cells. Vitr. Cell. Dev. Biol. Anim..

[43] Shirai Y., Okugawa Y., Hishida A., Ogawa A., Okamoto K., Shintani M., Morimoto Y., Nishikawa R., Yokoe T., Tanaka K. (2017). Fish oil-enriched nutrition combined with systemic chemotherapy for gastrointestinal cancer patients with cancer cachexia. Sci. Rep..

[44] Sun Y., Jia X., Hou L., Liu X., Gao Q. (2017). Involvement of apoptotic pathways in docosahexaenoic acid-induced benefit in prostate cancer: Pathway-focused gene expression analysis using RT2 Profile PCR Array System. Lipids Health Dis..

[45] Wang L., Choi H.S., Lee B., Choi J.H., Jang Y.-S., Seo J.-W. (2021). 13*R*,20-Dihydroxydocosahexaenoic Acid, a Novel Dihydroxy- DHA Derivative, Inhibits Breast Cancer Stemness through Regulation of the Stat3/IL-6 Signaling Pathway by Inducing ROS Production. Antioxidants.

[46] Wu Z., Chen C.Y., Kao C.L., Jiang Y., Liu C.M. (2019). Docosahexaenoic acid inhibits lipopolysaccharide-induced metastatic activities by decreasing inflammation on prostate cancer cell. Pharmazie.

[47] Yue H., Tian Y., Zhao Z., Bo Y., Guo Y., Wang J. (2022). Comparative Study of Docosahexaenoic Acid with Different Molecular Forms for Promoting Apoptosis of the 95D Non-Small-Cell Lung Cancer Cells in a PPARγ-Dependent Manner. Mar. Drugs.

[48] Zhang J., Xue B., Du C., Zhang L., Wang Y., Zhang Y., Li J. (2021). Docosahexaenoic acid supresses breast cancer cell proliferation and migration by promoting the expression of miR-99a and targeting mTOR signaling. Arab. J. Chem..

